# Preoperative Patient Expectation of Discharge Planning is an Essential Component in Total Knee Arthroplasty

**DOI:** 10.1186/s43019-022-00152-4

**Published:** 2022-05-08

**Authors:** James E. Feng, Afshin A. Anoushiravani, Jessica S. Morton, William Petersen, Vivek Singh, Ran Schwarzkopf, William Macaulay

**Affiliations:** 1 Department of Orthopedic Surgery, NYU Langone Health, 301 E 17th St., New York, NY 10003 USA; 2 Department of Orthopedic Surgery, Beaumont Health Royal Oak, Royal Oak, MI USA; 3 Department of Orthopaedic Surgery, Albany Medical Center, Albany, NY USA

**Keywords:** Total knee arthroplasty, Patient outcomes, Patient satisfaction, Shared decision-making, Length of stay, Joint replacement

## Abstract

**Purpose:**

A better understanding of total knee arthroplasty (TKA) candidate expectations within the perioperative setting will enable clinicians to promote patient-centered practices, optimize recovery times, and enhance quality metrics. In the current study, TKA candidates were surveyed pre- and postoperatively to elucidate the relationship between patient expectations and length of stay (LOS).

**Material and methods:**

This is a prospective study of patients undergoing TKA between December 2017 and August 2018. Patients were electronically administered surveys regarding their discharge plan 10 days pre-/postoperatively. All patients were categorized into three cohorts based on their LOS: 1, 2, and 3+ days. The effect of preoperative discharge education on patient postoperative satisfaction was evaluated.

**Results:**

In total, 221 TKAs were included, of which 83 were discharged on postoperative day (POD) 1, 96 on POD-2, and 42 POD-3+. Female gender, increasing body mass index (BMI), and surgical time correlated with increased LOS. Preoperative discussions regarding LOS occurred in 84.62% (187/221) of patients but did correlate with differences in LOS. However, patients discharged on POD-1 were more inclined to same-day surgery preoperatively. Patients discharged on POD-3+ were found to be more uncomfortable regarding their discharge during the preoperative phase. Multivariable regressions demonstrated that preoperative discharge discussion was positively correlated with home discharge.

**Conclusion:**

Physician-driven discussion regarding patient discharge did not alter patient satisfaction or length of stay but did correlate with improved odds of home discharge. These findings underscore the importance of patient education, shared decision-making, and managing patient expectations.

## Background

Total knee arthroplasty (TKA) is one of Medicare’s largest contributors to surgical expenditures—from 2002 to 2013, TKA total hospital costs tripled to $12 billion [1]. During this time period, average hospital costs per TKA were reported to increase only 52.4% ($7849) within the same time period [1]. The increase in total hospital costs has therefore been attributed to its rapid growth in overall demand and utilization [2–4]. As reported by the Healthcare Cost and Utilization Project (HCUP), it has been estimated that 700,100 inpatient TKAs were performed in 2012 alone, sustaining its position as the most common inpatient surgical procedure in the USA [5]. Despite a recent slowing in TKA volume, it is estimated that 935,000 TKAs will be performed per annum by 2030 [4].

Under the current payment models, healthcare organizations have shifted their focus from volume-based care to a system of value-based and quality-driven care [6–11]. In doing so, substantially greater emphasis has been placed on improving hospital operating efficiency, improving patient safety and satisfaction, and reducing care variability. One specific area of focus has been securing timely discharges as an initiative to reduce per-episode-of-care expenditures for TKA [6–11].

Recent literature has emphasized the value of shared decision-making with preoperative patients as a model to educate patients about their perioperative care, improve patient satisfaction, and reduce episode-of-care costs. The “DECISIONS” study by Zikmund-Fisher et al. highlighted the need for orthopedic surgeons to better understand, communicate, and manage patient expectations in the perioperative setting [12]. For orthopedic surgeons, indications for TKA were discussed in only 76% of patient encounters, and patient treatment preferences were discussed in only 72% of surgical cases [12]. As medicine continues to transition away from its paternalistic roots and incentivizes a more patient-centric approach, patient education and the incorporation of patient care preferences will be paramount [12].

In a previous multicenter study evaluating patient satisfaction following total hip arthroplasty (THA), physician-initiated discussion of patient discharge was correlated with shorter lengths of stay (LOS) and improved patient satisfaction [13]. By implementation of Standardized Care Pathways (SCPs) set forth by the Centers for Medicare and Medicaid Services (CMS), institutions have successfully reduced the LOS while maintaining the current quality of care; however, there continues to be a paucity of studies evaluating the effects of SCPs on patient-perceived effects, expectations, and satisfaction [6, 7]. Patient education and expectations are common components of many of the current pathways and education programs and have encountered a wide range of results [14–17]. Furthermore, as patient-reported outcomes (PROs) and the Hospital Consumer Assessment of Healthcare Providers and Systems (HCAHPS) scores become an integral component of future alternative payment models (APMs), the importance of patient satisfaction and shared decision-making becomes synonymous with healthcare organization and physician reimbursement [8–11, 18].

The primary objective of our study is to evaluate the effect of preoperative, physician-initiated shared decision-making regarding hospital LOS and its effect on postoperative patient satisfaction for patients undergoing TKA. We hypothesize that preoperative shared discussion will enhance postoperative patient satisfaction and outcomes.

## Materials and methods

This is a prospective observational study of TKA candidates between December 2017 and August 2018 at a single, urban, academic healthcare organization. As part of our institution’s standard of care practices, all patients scheduled for total joint arthroplasty were enrolled in a commercially available electronic patient rehabilitation application (EPRA) (Force Therapeutics; New York, NY). Briefly, EPRA is a digital, customizable episode-of-care management tool designed to provide patients with pre- and postoperative educational materials in the form of text, videos, and short quizzes, while also acting as a communication portal between patients and healthcare providers. Moreover, the EPRA platform can push and email custom and validated patient-reported outcome (PRO) surveys to the patient’s smart device or computer, allowing physicians to better track the progress of their patients. Given the platform capabilities, we designed a novel survey to assess the effects of managing patient expectations on patient discharge satisfaction, as assessed by patient-reported “comfort” (Table 1).

**Table 1:** Questions

Preoperative questions
Did your surgeon discuss the duration of your hospital stay?
Yes
No
How long was the estimated duration?
Same-day discharge (no overnight)
Next day (overnight)
2 days
3+ days
N/A (my surgeon did not discuss my length of stay)
How comfortable are you with this discharge plan?
Very comfortable
Somewhat comfortable
Somewhat uncomfortable
Very uncomfortable
If offered, would you be willing to participate in a same-day-surgery program,
Very likely
Likely
Unlikely
Very unlikely
Postoperative questions
What day were you discharged from surgery?
Same-day discharge (no overnight)
Next day (overnight)
2 days
3+ days
How comfortable were you with your discharge from the hospital?
Very comfortable
Somewhat comfortable
Somewhat uncomfortable
Very uncomfortable
Would you have preferred to be discharged earlier?
Yes, a day or two earlier
No, I was satisfied with my discharge
No, preferred to stay longer

All patients were electronically administered surveys regarding their discharge planning 10 days pre- and postoperatively via push notifications and emails. Patient demographics, surgical factors, and hospital-reported LOS were queried from our institution’s electronic data warehouse, Epic Caboodle (Verona, WI), utilizing Microsoft SQL Server Management Studio (Redmond, WA). Missing patient data were manually chart-checked via our electronic health record (EHR) system, Epic Hyperspace (Verona, WI). Patients were categorized into three cohorts on the basis of their actual hospital LOS based on calendar day: 1 day, 2 day, and 3+ days. Patients discharged on postoperative day (POD) 0 were excluded from the study owing to limited sample size (*n* = 6) but retained in our tables for descriptive purposes only. It was noted that two surgeons had only contributed a single patient each to the study. To minimize potential bias, their respective two patients were excluded from the study. The remaining surgeons contributed equally to the study (range 44–63 patients each).

All data transformations and statistical analyses were performed using the Anaconda (version 5.3.0; Anaconda Inc., Austin, TX) distribution of Python (version 3.6.6; Python Software Foundation, https://www.python.org). Libraries utilized in this study included *pandas*, *numpy*, *scipy*, *statsmodels*, *patsy*, and their respective library dependencies. Statistical analyses of univariable continuous outcomes were performed utilizing one-way analysis of variance (ANOVA) testing, while categorical variables were tested utilizing *χ*^2^ tests. Multivariable logistic regression was performed to evaluate what factors effect patient discharge comfort, as well as a separate multivariable linear regression to evaluate what factors may affect actual inpatient LOS. A *p*-value of < 0.05 was deemed significant.

## Results

### Patient demographics

In total, 221 elective, primary TKAs were performed by four surgeons within the study period (Table 1). Eighty-three (37.56%) TKAs were discharged on POD-1, 96 (43.44%) were discharged on POD-2, and 42 (19.00%) were discharged on POD-3 or later. Assessment of patient demographics demonstrated that female gender (female gender prevalence: POD-1 discharge 48.19%, POD-2 discharge 62.50%, POD-3+ discharge 76.19%; *p* < 0.01), increasing BMI [average BMI ± 1 standard deviation (SD): POD-1 discharge 30.09 ± 6.24 kg/m^2^, POD-2 discharge 31.27 ± 6.59 kg/m^2^, POD-3+ discharge 33.88 ± 8.25 kg/m^2^; *p* < 0.05] and insurance type (commercial versus Medicare insurance: POD1 discharge 56.63% versus 39.76%, POD2 discharge 55.21% versus 36.46%, POD1 discharge 28.57% versus 57.14) were correlated with longer LOS cohorts. While nonsignificant, patients who were categorized as “single, divorced, or widowed” were more prevalent in extended LOS cohorts (POD-1 discharge 43.37%, POD-2 discharge 41.67%, POD-3+ discharge 61.90%; *p* = 0.07). Age (*p* = 0.91), race (*p* = 0.83), smoking status (*p* = 0.53), and surgical time (*p* = 0.26) were similar between cohorts (Table 2).

**Table 2:** Baseline demographics

Demographics	Actual length of stay				*p*	Aggregated *n* = 221^a^
	0 *n* = 6*	1 *n* = 83	2 *n* = 96	3 + *n* = 42		
Age (years)	54.00 ± 9.25	62.01 ± 10.47	61.65 ± 10.33	62.45 ± 11.43	0.92	61.94 ± 10.55
Gender					< 0.01	
Female	3 (50.00%)	40 (48.19%)	60 (62.50%)	32 (76.19%)		132 (59.73%)
Male	3 (50.00%)	43 (51.81%)	36 (37.50%)	10 (23.81%)		89 (40.27%)
BMI (kg/m^2^)	29.68 ± 6.00	30.09 ± 6.24	31.27 ± 6.59	33.88 ± 8.25	< 0.05	31.28 ± 6.85
Race					0.83	
African American (Black)	0	11 (13.25%)	19 (19.79%)	9 (19.05%)		38 (17.19%)
Asian	0	2 (2.41%)	3 (3.12%)	0		5 (2.26%)
White	6 (100.00%)	62 (74.70%)	65 (67.71%)	30 (71.43%)		157 (71.04%)
Unknown	0	8 (9.64%)	9 (9.38%)	4 (9.52%)		21 (9.50%)
Smoking status					0.53	
Current smoker	0	5 (6.02%)	7 (7.29%)	3 (7.14%)		15 (6.79%)
Former smoker	3 (50.00%)	31 (37.35%)	38 (39.58%)	22 (52.38%)		91 (41.18%)
Never	3 (50.00%)	47 (56.63%)	51 (53.12%)	17 (40.48%)		115 (52.04%)
Primary payor					< 0.05	
Commercial	5 (83.33%)	47 (56.63%)	53 (55.21%)	12 (28.57%)		112 (50.68%)
Medicaid	0	3 (3.61%)	7 (7.22%)	4 (9.52%)		14 (6.33%)
Medicare	1 (16.67%)	33 (39.76%)	35 (36.46%)	24 (55.81%)		92 (41.63%)
Worker’s compensation/no fault	0	0	1 (1.04%)	2 (4.76%)		3 (1.36%)
Marital status					0.07	
Married/partner	5 (83.33%)	47 (56.63%)	56 (58.33%)	16 (38.10%)		119 (53.85%)
Single/divorced/widowed	1 (16.67%)	36 (43.37%)	40 (41.67%)	26 (61.90%)		102 (46 .15%)
Surgical time (min)	118.50 ± 26.26	94.29 ± 18.82	98.22 ± 25.56	101.67 ± 32.52	0.27	97.40 ± 24.87
Discharge disposition					< .0001	
Home with self-care	1 (83.33%)	4 (4.82%)	2 (2.08%)	1 (2.38%)		7 (3.17%)
Home with services	5 (16.67%)	78 (93.98%)	92 (95.83%)	30 (71.43%)		200 (90.50%)
Skilled nursing facility	0	1 (1.20%)	2 (2.08%)	11 (26.19%)		14 (6.33%)

^a^Not included in statistical analyses, and excluded from aggregated column

### Survey validity and administration

To assess our patient population’s understanding of their inpatient stay, patients were asked the duration of their LOS in a postoperative survey and these values were compared with their actual LOS (Table 3). Patient-reported LOS was correct in 96.38% of surveys (21/221) patients; denoted by ^‡^ in Table 3). All surveys were provided 10 ± 9 days before and 9 ± 2 days after their day of discharge.

**Table 3:** Survey

	Length of stay				*p*	Aggregated *n* = 221*
	0 *n* = 6*	1 *n* = 83	2 *n* = 96	3 + *n* = 42		
Preoperative						
LOS discussion					0.57	
Yes	6 (100.00%)	68 (81.93%)	84 (87.50%)	35 (83.33%)		187 (84.62%)
No	0	15 (18.07%)	12 (12.37%)	7 (16.28%)		34 (15.38%)
Estimated LOS					NA	
Same day	**5 (83.33%)**	3 (3.61%)	1 (1.04%)	2 (4.76%)		6 (2.71%)
Next day	0	**35 (42.17%)**^†^	27 (27.12%)	3 (7.14%)		65 (29.41%)
2 days	1 (16.67%)	30 (36.14%)	**49 (51.04%)**^**†**^	21 (50.00%)		100 (45.25%)
3+ days	0	3 (3.61%)	10 (10.42%)	**10 (23.81%)**		23 (10.41%)
Not discussed	0	12 (14.46%)	9 (9.38%)	6 (14.29%)		27 (12.22%)
Discharge plan					< 0.01	
Very comfortable	3 (50.00%)	46 (55.42%)	39 (40.62%)	12 (28.57%)		97 (43.89%)
Somewhat comfortable	3 (50.00%)	28 (33.73%)	44 (45.38%)	16 (38.10%)		88 (39.82%)
Somewhat uncomfortable	0	5 (6.02%)	6 (6.52%)	9 (21.43%)		20 (9.05%)
Very uncomfortable	0	4 (4.82%)	7 (7.29%)	5 (11.90%)		16 (7.24%)
Likelihood to participate in SDD					< .0001	
Very likely	3 (50.00%)	20 (20.62%)	7 (7.29%)	3 (7.14%)		30 (13.57%)
Likely	2 (33.33%)	32 (38.55%)	20 (20.83%)	3 (7.14%)		55 (24.89%)
Unlikely	1 (16.67%)	21 (25.30%)	36 (37.50%)	12 (28.57%)		69 (31.22%)
Very unlikely	0 (0.00%)	10 (12.05%)	33 (34.38%)	24 (57.14%)		67 (30.32%)
Postoperative						
Patient-reported LOS					NA	
Same day	**6 (100.00%)**	0	0	0		0
Next day	0	**82 (98.80%)**^‡^	3 (3.12%)	0		85 (38.46%)
2 days	0	1 (1.20%)	**91 (94.79%)**	2 (4.76%)		94 (42.53%)
3+ days	0	0	2 (2.08%)	**40 (95.24%)**		42 (19.00%)
Discharge comfort					0.09	
Very comfortable	3 (50.00%)	50 (60.24%)	52 (54.17%)	17 (40.48%)		119 (53.85%)
Somewhat comfortable	0	24 (28.92%)	36 (37.50%)	19 (45.24%)		79 (35.75%)
Somewhat uncomfortable	3 (50.00%)	6 (7.23%)	8 (8.33%)	6 (14.29%)		20 (9.05%)
Very uncomfortable	0	3 (3.61%)	0	0		3 (1.36%)
Preferred earlier discharge					0.12	
Preferred a longer stay	2 (33.33%)	9 (10.84%)	11 (11.46%)	10 (23.81%)		30 (13.57%)
Satisfied with stay	4 (66.67%)	74 (89.16%)	82 (85.42%)	31 (73.81%)		187 (84.62%)
Preferred a day or two earlier	0	0	3 (3.12%)	1 (2.38%)		4 (1.81%)

^*^Not included in statistical analyses, and excluded from aggregated column

^†^Correctly estimated LOS were provided to patients as assessed by preoperative survey

^‡^Patient correctly reported length of stay when compared with hospital-recorded length of stay

### Preoperative survey

Discussions regarding a patient’s LOS was reported in 84.62% (187/221 patients) of preoperative patient encounters and was equally distributed among the cohorts (*p* = 0.57). With regard to preoperative estimated LOS, as reported by patients, only 42.53% (94/221 patients; denoted by ^†^ in Table 3) were provided accurate estimates of their expected LOS.

Across all cohorts, 43.98% of patients reported feeling “very comfortable,” and 39.82% of patients reported feeling “somewhat comfortable,” with the preoperative discharge plans. Moreover, patients in cohorts with longer LOS were less likely to feel “very comfortable” with their discharge plan preoperatively [“very comfortable”: POD-1 discharge 55.42% (46/83 patients), POD-2 discharge 40.62% (39/96 patients), POD-3+ discharge 28.57% (12/42 patients); < 0.01], and were also less likely to have enrolled in a same-day discharge (SDD) program if provided the opportunity [“very likely” or “likely”: POD 1 discharge 62.65% (52/83 patients), POD-2 discharge 28.13% (27/96 patients), POD-3+ discharge 14.29% (6/42 patients); < 0.0001].

### Postoperative survey

Postoperatively, patients generally reported an increase in their comfort with their discharge planning (Table 3). In total, 53.85% (119/221 patients) of patients reported being “very comfortable,” up from the 43.89% preoperatively. Meanwhile, only 1.36% of patients felt very uncomfortable regarding their discharge, substantially less than the 7.24% preoperatively. Cohorts with longer LOS were less likely to elicit “very comfortable” responses [“very comfortable”: POD-1 discharge 60.24% (50/83 patients), POD-2 discharge 54.17% (52/96 patients), POD-3+ discharge 40.48% (17/42 patients)], but these findings were not statistically significant (*p* = 0.09). When surveyed about LOS duration, 84.62% (187/221) of patients were satisfied, similar among the groups (*p* = 0.13).

### Preoperative factors affecting discharge

To evaluate whether preoperative discharge discussions improved patient comfort at discharge, a *χ*^2^ test was performed, which was nonsignificant (*p* = 0.39). A follow-up multivariable logistic regression was performed (Table 4). Postoperative patient-reported comfort with their discharge plan was separated into binary outcomes: “comfortable” and “uncomfortable.” No significant correlation was found between preoperative discharge discussion and postoperatively surveyed discharge comfort (*p* = 0.80).

**Table 4:** Evaluating the effects of preoperative discussion regarding patient LOS and overall comfort with a patient’s discharge

Variable	Odds ratio (95% CI)	*p*
Preoperative discussion		
No	**Ref.**	–
Yes	**2.73 (0.90–8.25)**	**0.80**
Age	1.00 (0.94–1.06)	0.87
BMI	1.00 (0.93–10.7)	0.98
Gender		
Female	Ref.	–
Male	1.52 (0.54–4.30) 1.79 (0.60–5.33)	0.43 0.30
Race		
White	Ref.	–
African American	7.74 (0.30–198.17)	0.22
Asian	3.23 (0.37–28.25)	0.22
Unknown	1.46 (0.13–16.54)	0.76
Smoking status		
Never smoker	Ref.	–
Former smoker	0.45 (0.04–4.66)	0.44
Current smoker	0.54 (0.21–1.43)	0.16
Marital status		
Married/partner	Ref.	–
Single/divorced/widowed	0.52 (0.20–1.34) 0.48 (0.18–1.26)	0.18
Insurance type		
Commercial		–
Medicare	2.80 (0.36–22.07)	0.29
Medicaid	1.87 (0.54–6.52)	0.58
Worker’s compensation	14.80 (0.73–300.72)	0.11
Length of stay (days)	0.86 (0.49–1.50)	0.65

Discharge comfort was derived from each patient’s postoperative survey. To satisfy the requirements of a binary outcome for the logistic regression, very comfortable and somewhat comfortable were transformed into “comfortable,” while somewhat uncomfortable and very uncomfortable were transformed into “uncomfortable”

A secondary multivariable linear regression analysis of patient factors affecting inpatient LOS was performed (Table 5). The presence or absence of a preoperative discussion was not found to significantly alter inpatient LOS (*β* = −0.01; 95% CI 0.30–2.42; *p* = 0.95). Conversely, male gender (*β* = 0.34; 95% CI 0.10–0.59; *p* < 0.05), Medicare payor type (*β* = 0.56; 95% CI 0.06–1.05; *p* < 0.001), and worker’s compensation/no-fault payor type (*β* = 1.75; 95% CI 0.80–2.71; *p* < 0.001) were all significantly correlated with increased inpatient LOS to varying degrees. Current smoker was correlated with a shorter length of stay (*β* = −0.24; 95% CI −0.47 to 0.01; *p* < 0.05).

**Table 5:** Evaluating the effects of preoperative discussion regarding patient LOS and actual LOS

Variable	Coefficient (95% CI)	*p*
Preoperative discussion		
No	**Ref.**	
Yes	**0.01 (**−**0.30 to 2.42)**	**0.95**
Age	−0.00 (−0.02 to 0.03)	0.64
BMI	0.01 (−0.00 to 0.03)	0.10
Gender		
Female	Ref.	
Male	0.34 (0.10 to 0.58)	< 0.01
Race		
White	Ref.	
African American	−0.11 (−0.92 to 0.70)	0.78
Asian	0.09 (−0.30 to 0.48)	0.65
Unknown	0.08 (−0.38 to 0.54)	0.74
Smoking status		
Never smoker	Ref.	
Former smoker	−0.22 (−0.70 to 0.25)	0.35
Current smoker	−0.24 (−0.47 to −0.01)	< 0.05
Marital status		
Married/partner	Ref.	
Single/divorced/widowed	−0.10 (−0.33 to 0.14)	0.41
Insurance type		
Commercial	Ref.	
Medicare	0.56 (0.06 to 1.05)	< 0.05
Medicaid	0.24 (−0.05 to 0.52)	0.11
Worker’s compensation	1.75 (0.80 to 2.71)	< 0.001

Finally, a multivariable logistic regression evaluating the effect of preoperative discharge discussions on discharge disposition was performed (Table 6). As 98.19% (217/221) patients were discharged home, regression convergence was not possible for race, smoking status, and insurance type. Race, smoking, and worker’s compensation/no fault (three patients) were excluded from the analysis. Preoperative discussions were found to significantly increase likelihood of home discharge compared with a skilled nursing facility (OR 3.87; 95% CI 1.05–14.20; *p* < 0.05). Similarly, increasing age was correlated with increased odds for home discharge (OR 1.07; 95% CI 1.00–1.15; *p* < 0.05). Conversely, single, divorced, or widowed marital status was correlated with increased odds of subacute nursing facility discharge (OR 0.24; 95% CI 0.06–094; *p* < 0.05).

**Table 6:** Evaluating the effects of preoperative discussion regarding patient discharge disposition to home

Variable	Odds ratio (95% CI)	*p*
Preoperative discussion		
No	**Ref.**	
Yes	**3.87 (1.05–14.20)**	** < 0.05**
Age	1.07 (1.00–1.15)	< 0.05
BMI	1.06 (0.97–1.15)	0.20
Gender		
Female	Ref.	
Male	2.17 (0.48–9.76)	0.31
Marital status		
Married/partner	Ref.	
Single/divorced/widowed	0.24 (0.06–0.94)	< 0.05

## Discussion

TKA remains a major driver of Medicare surgical expenditures [1], and length of stay is a major driver of inpatient costs [19, 20]. Nationally, implementation of Standardized Care Pathways (SCPs) set forth by CMS has resulted in decreased LOS for patients undergoing TKA [6, 7]. Care pathways are an evidence-based multidisciplinary approach that includes preoperative education; these pathways have been implemented with success and reductions in hospital expenditures by several groups [6, 21, 22]. Patient-reported outcomes, especially patient satisfaction metrics, are increasingly being incorporated into reimbursement models as metrics of success. Physicians’ ability to influence patient expectations of postoperative course and disposition may be a means to not only improve reimbursement and metrics of success but better engage patients and increase comfort with their care. In our study, 83% of patients were very comfortable or somewhat comfortable with their preoperative discharge plan, which increased to 89% being very comfortable or somewhat comfortable postoperatively. Eighty-four percent of patients received preoperative discussion regarding length of stay and discharge disposition. This is higher than previously published in reports of orthopedic surgeons [12].

Despite the initiation of preoperative counseling, it remains difficult to accurately inform patients of their exact LOS. Our data demonstrate that 42.5% (9/221 patients) were provided accurate estimates of their expected LOS. In a recent meta-analysis, Shah et al. found increasing age, female gender, BMI ≥ 30 kg/m^2^, and American Society of Anesthesiology (ASA) score > 2 to increase LOS [23]. In our study, female gender and worker’s compensation insurance were associated with increased length of stay on multivariate analysis. Female gender has been associated with increased length of stay by several authors [24–26]. Proposed explanations include increased pain and increased severity of arthritis prior to intervention and decreased social support at home. The number of patients with the primary payor of worker’s compensation or no fault included in this study was small, only three of the total 221 patients and likely not generalizable. Worker’s compensation claim status has been a risk factor for decreased satisfaction in other orthopedic conditions [27–29].

In this prospective observational study, preoperative discussions regarding patient discharge nonsignificantly trended toward improved patient comfort toward discharge during the postoperative phase, but there was no perceivable effect on LOS. The effect of preoperative education in knee replacement was examined in a Cochrane review in 2014 by McDonald et al., who at that time found an almost 2-day reduction in length of stay for patients undergoing TKA who received preoperative education [30]. Pamilo et al. also found a significant decrease in length of stay and an increased proportion of discharges to home without an increase in revisions, manipulations, mortality, or readmissions [31]. While patients were counseled by both a surgeon and nurse, it is presumed that an institution-wide adoption of a “fast track” program was the primary driver of reduced LOS. Husted and colleagues stressed the importance of counseling both the patient and their family, providing both written and verbal information regarding length of stay when implementing and organizing a fast-track program [25, 32]. We therefore hypothesize that an institutional adoption of patient counseling regarding discharge is more effective than surgeon-driven counseling in isolation.

When compared with commercial payor types, Medicare and worker’s compensation/no fault was significantly correlated with increasing LOS. In a case–control by Halawi et al., Medicare insurance type was significantly correlated with 4.42 greater odds for inpatient LOS > 2 days when compared with commercial payor types, which was attributed primarily to the CMS’s inpatient-only status of TKA at the time of their study [33]. The effect is further substantiated when compared with Medicaid payor types, which demonstrated 3.88 greater odds for > 2 days LOS for Medicare patients.

Smoking was correlated with a shorter LOS by 0.24 days when compared with nonsmokers on multivariable linear regression. A previous study at our institution demonstrated a 0.15-day (2.47 versus 2.62 days; *p* = 0.56) decrease in LOS for patients who completed our 4–6-week preoperative smoking cessation program when compared with continued smokers [34]. In a randomized control trial in Denmark by Moller et al., smoking cessation was also demonstrated to decrease inpatient length of stay (11 days versus 13 days) [35].

In recent years, the LOS for TKA has declined from weeks to days [1]; in our study, most patients were discharged on POD-1 or 2. Further reductions in LOS may have diminishing financial returns. However, preoperative discussions do help set patient expectations and identify patients with barriers to discharge, including reduced social support [32]. Discharge destination impacts both quality measures and cost. Our study found that patients receiving preoperative discussion were significantly more likely to be discharged to home than a subacute nursing facility. In a retrospective review of 372 patients undergoing consecutive total joint arthroplasty, Halawi and colleagues found that age, caregiver support at home, and patient expectation of discharge destination were the only significant predictors of discharge destination. Among the variables examined, patient expectation was the most important predictor (*p* < 0.001). Their group has begun to incorporate discharge destination determination in the preoperative clinic visit leading to a subjective appreciation of decreased LOS [36]. Patients’ readiness for discharge is a complex interplay of physiologic, psychologic, and social factors [37]. In a study of total hip arthroplasty (THA) recipients, Heine et al. found the main concern of patients prior to discharge was feeling safe at home, which included both patients’ personal confidence in their abilities and support of family [38]. Both of these components may be addressed pre- and postoperatively. Our study assessed feelings of comfort with the discharge plan; while most patients were very comfortable or somewhat comfortable, a proportion of patients noted a level of discomfort in their plan. Targeted intervention regarding the lack of comfort in these patients may provide a method to decrease the length of stay and increase patient satisfaction.

Paradoxically, older patients in our study demonstrated an increased rate of home discharge based on our multivariable linear regression. This contradicts previous studies demonstrating that older and more geriatric patients are more likely to experience longer LOS and discharge to skilled nursing facilities [39]. At our institution, discharge to skilled nursing facilities is mostly secondary to limited social support (living alone) and/or home environmental factors, particularly walkups and stairs. It is therefore hypothesized that our increased discharge rate to facilities in comparably younger patients may be skewed by factors such as younger patients living alone and/or in higher walkup housing, while older patients may have accommodated themselves in homes with limited stairs, elevator access, and/or living with family support or in assistive living. It is also conceivable our results may be the result of a type II error.

We found patients who were single or divorced trended toward discharge to a facility rather than home. Literature regarding the association of social support and discharge destination is mixed. Slover et al. found no association between pain-catastrophizing behavior or social support with length of stay or discharge disposition [26]. Weaver et al. found that females undergoing TKA were less likely to be married than their male counterparts, and not being married was associated with longer LOS in both males and females [40]. Napier et al. cited social reasons as the most common cause for delayed discharge in patients undergoing THA or TKA and recommended preoperative agreement regarding discharge plan prior to admission for arthroplasty [41]. As marital status remains a proxy for social support, surgeons and the multidisciplinary teams involved in planning patients’ preoperative discharge destination should inquire about the patient’s social setting to ensure safe and timely discharge.

The relationship between shared decision-making and measurable quality improvement is difficult to objectively assess within the clinical setting. Our investigation did not find a significant reduction in LOS among patients having preoperative discharge discussions. However, these patients were more likely to be discharged home with improved patient comfort. Physicians and other members of the care team should be encouraged to discuss LOS and discharge disposition with TKA candidates with the goal of improving patient satisfaction, readiness for discharge, and the preoperative identification of any healthcare barriers.

## Conclusion

While the results of our study indicate that physician-driven discussion regarding patient discharge does not alter patient satisfaction or length of stay, it did correlate with improved odds of home discharge. These findings underscore the importance of patient education, shared decision-making, and managing patient expectations. Future studies are needed to evaluate what key components of the physician–patient interaction may address underlying patient anxieties and/or hesitations for shorter LOS and home discharge. Additional studies investigating potential financial savings and the economic impact of these simple physician–patient interactions are also warranted.

## Data Availability

The datasets generated during and/or analyzed during the current study are available from the corresponding author on reasonable request.

## Abbreviations

TKA: Total knee arthroplasty
THA: Total hip arthroplasty
HCUP: Healthcare Cost and Utilization Project
LOS: Length of stay
SCPs: Standardized Care Pathways
CMS: Centers for Medicare and Medicaid Services
PROs: Patient-reported outcomes
HCAHPS: Hospital Consumer Assessment of Healthcare Providers and Systems
APM: Alternative payment models
EPRA: Electronic patient rehabilitation application
EHR: Electronic health records
SDD: Same-day discharge
POD: Postoperative day
